# Decitabine Sensitizes the Radioresistant Lung Adenocarcinoma to Pemetrexed Through Upregulation of Folate Receptor Alpha

**DOI:** 10.3389/fonc.2021.668798

**Published:** 2021-05-17

**Authors:** Yuqing Wang, Jie Huang, Qiong Wu, Jingjing Zhang, Zhiyuan Ma, Lucheng Zhu, Bin Xia, Shenglin Ma, Shirong Zhang

**Affiliations:** ^1^ Translational Medicine Research Center, Key Laboratory of Clinical Cancer Pharmacology and Toxicology Research of Zhejiang Province, Affiliated Hangzhou First People’s Hospital, Zhejiang University School of Medicine, Cancer Center, Zhejiang University, Hangzhou, China; ^2^ Department of Oncology, Affiliated Hangzhou Cancer Hospital, Zhejiang University School of Medicine, Hangzhou, China; ^3^ The Fourth College of Clinical Medicine, Zhejiang Chinese Medical University, Hangzhou, China; ^4^ Department of Cancer Medical Center, Affiliated Xiaoshan Hospital, Hangzhou Normal University, Hangzhou, China

**Keywords:** lung adenocarcinoma (LUAD), radioresistance, pemetrexed, folate receptor α, decitabine

## Abstract

Chemotherapy is the backbone of subsequent treatment for patients with lung adenocarcinoma (LUAD) exhibiting radiation resistance, and pemetrexed plays a critical role in this chemotherapy. However, few studies have assessed changes in the sensitivity of LUAD cells to pemetrexed under radioresistant circumstances. Therefore, the objectives of this study were to delineate changes in the sensitivity of radioresistant LUAD cells to pemetrexed and to elucidate the related mechanisms and then develop an optimal strategy to improve the cytotoxicity of pemetrexed in radioresistant LUAD cells. Our study showed a much lower efficacy of pemetrexed in radioresistant cells than in parental cells, and the mechanism of action was the significant downregulation of folate receptor alpha (FRα) by long-term fractionated radiotherapy, which resulted in less cellular pemetrexed accumulation. Interestingly, decitabine effectively reversed the decrease in FRα expression in radioresistant cells through an indirect regulatory approach. Thereafter, we designed a combination therapy of pemetrexed and decitabine and showed that the activation of FRα by decitabine sensitizes radioresistant LUAD cells to pemetrexed both *in vitro* and in xenografts. Our findings raised a question regarding the administration of pemetrexed to patients with LUAD exhibiting acquired radioresistance and accordingly suggested that a combination of pemetrexed and decitabine would be a promising treatment strategy.

## Introduction

Radiotherapy has been widely administered to patients with local lung adenocarcinoma (LUAD) patients to eliminate cancer cells (1). However, a majority of patients finally progress after radiation treatment due to either intrinsic insensitiveness or acquired radioresistance, which accelerates aggressive tumor growth or metastasis and further causes poor survival (2). Pemetrexed-based chemotherapy is currently commonly used as the first-line and second line treatment strategy for patients with recurrent or metastatic lung adenocarcinoma either alone or in combination with antiangiogenic drugs or immune checkpoint blockers, as recommended in various guidelines (3, 4). However, a prospective phase II study (5), which combined low-dose radiotherapy and pemetrexed in the treatment of recurrent NSCLC patients, found only 20% of all the previously irradiated patients showed a partial response, which suggested these previously irradiated tumor cells might have resistance to pemetrexed. Another clinic trial treated locally advanced NSCLC with carboplatin/pemetrexed plus radiotherapy and found three patients (20%) without distant metastases at registration developed distant metastases at a median time of 5 months, although these patients received consolidation carboplatin/pemetrexed for 2-3 cycles (6). These data suggested long-term fractionated radiotherapy may change the sensitivity of NSCLC cells to pemetrexed.

Radioresistant cancer cells display distinct gene-phenotypes with higher DNA repair and antioxidant capacities than their parental cells because of the complicated and repeated DNA damage repair process that occurs during the long-term radiation treatment (7, 8) and the dormancy-repopulation cycle of radioresistant cancer stem cells in the acidic tumor microenvironment (9), which may substantially alter the efficacy of chemotherapeutic drugs. For instance, radioresistant MDA-MB-231 breast cancer cells are resistant to paclitaxel (10). Radioresistant esophageal squamous cancer cells are resistant to paclitaxel through the upregulation of P-glycoprotein (11). Whereas, our recent study indicated that radioresistant LUAD cells showed increased sensitivity to SN-38, paclitaxel and docetaxel (12). However, the efficacy of pemetrexed on radioresistant LUAD cells was unclear and there were no basic studies, clinical trials or retrospective studies focused on this scenario. Thus, studies of changes in the cytotoxicity of pemetrexed toward radioresistant cancer cells are necessary.

Pemetrexed is a multitargeted antifolate drug that inhibits DHFR (dihydrofolate reductase), TYMS (thymidylate synthase), and GART (glycinamide ribonucleotide formyltransferase) in the folate pathway (13). Pemetrexed itself mainly relies on folate transporters and folate receptor proteins on the cell membranes to enter cells (13, 14), including reduced folate carrier (RFC), proton-coupled folate transporter (PCFT), folate receptor protein α (FRα), and folate receptor protein β (FRβ), of which FRα is expressed at high levels in epithelial cells of the placenta, female reproductive organs, breast, lung and various human solid tumors (15). FRα, as a high-affinity binding protein encoded by the gene FOLR1, mediates pemetrexed transport through endocytosis. Studies have indicated that patients with LUAD generally have high FRα expression levels (16–18), and the expression level of FRα directly affects the clinical efficacy of pemetrexed (19, 20). Therefore, FRα plays important role in the clinical outcomes of pemetrexed treatment. Interestingly, our study found most LUAD patients presented a significant reduction in serum FRα levels after receiving an average dose of more than 50 Gy radiotherapy, suggesting that long-term radiation treatment may change the expression of FRα and finally affect the efficacy of pemetrexed.

Given the importance of radiotherapy and pemetrexed in the clinical treatment of LUAD and the limited data on changes in FRα expression during long-term radiation treatment, the first aim of the present study was to delineate the altered sensitivity of radioresistant LUAD cells to pemetrexed based on the regulation of FRα. The secondary aim was trying to illustrate the potential regulatory mechanisms and develop a proper drug combination strategy that will increase the cytotoxicity of pemetrexed toward radioresistant lung adenocarcinoma.

## Materials and Methods

### Cell Culture and Reagents

NCI-H1975, NCI-H1650, NCI-HCC827, NCI-H1299, A549 and PC9 cell lines were purchased from the Type Culture Collection of the Chinese Academy of Sciences (Shanghai, China). They were maintained in RPMI-1640 medium (Gibco-Life Technologies, NY, USA) supplemented with 10% fetal bovine serum (Gibco-Life Technologies), 100 μg/mL streptomycin and 100 U/mL penicillin (Gibco-Life Technologies). Cells were cultured at 37°C with a humid atmosphere of 5% CO_2_ and 95% air. Pemetrexed, decitabine, and cycloheximide were obtained from Selleck Chemicals (Houston, TX, USA).

### Clinical Specimens

Surgical specimens from 18 patients with lung adenocarcinoma patients and paired serum samples collected within 3 days before and after radiotherapy alone from seven adenocarcinoma patients were obtained from Hangzhou First People’s Hospital, which was approved by the Institutional Review Board of Hangzhou First People’s Hospital. The relevant clinic pathological information of the enrolled patients was listed in **Supplementary Table 1**. All private information of each individual has been carefully blocked.

### Establishment of Radioresistant A549 Cells

Cell irradiation was performed with an X-RAD 225 irradiator provided by Precision (North Branford, USA). Briefly, a dose of 2 Gy (3.2 Gy/min, 13.3 mA, 225 kV, 2 mm Al filter) radiation was delivered to 50% confluent A549 cells at room temperature, and then the cells were maintained in culture. Cells were passaged to new flasks when they reached approximately 80% confluence. Afterwards, a repetitive 2 Gy dose of radiation was delivered to 50% confluent cells until a cumulative dose of 60 Gy was administered. A549 cells with acquired radioresistance were named A549R cells, and cells at passages 5-6 were used in all the subsequent experiments. The parental cells without irradiation were cultured and passaged simultaneously.

### Plasmid Constructs and Cell Transfection

For the knockdown of FRα, a short hairpin RNA was inserted into the lentiviral vector pLent-U6-GFP-Puro (Vigene Biosciences, Shandong, China). For FRα overexpression, the full-length FRα cDNA was inserted into the lentiviral vector pReceiver-Lv201 purchased from FulenGen Company (Guangzhou, China). Virus particles were packaged in HEK293T cells by transfection with Lipofectamine 3000 (Invitrogen, IL, USA). A549, A549R or H1299 cells were infected with the lentivirus-conditioned medium and 8 μg/mL polybrene (Sigma-Aldrich, MO, USA). Stably-transfected cells were screened by using puromycin (Sigma-Aldrich) at a concentration of 2.5 μg/mL. Target sequences of shRNAs are listed in **Supplementary Table 2**.

### RT-qPCR Analyses

Total RNA was extracted by using an AxyPrep Multisource RNA Miniprep Kit (Axygen, MA, USA) as described recently (12). The cDNA templates were synthesized from 500 ng of RNA using a commercially available reverse transcription kit (Takara, Japan). RT-qPCR was performed using a 7500 System (Applied Biosystems, Singapore) and TB Green Premix Ex Taq (Takara, Japan). All samples were analyzed in 3 biological replicates, with the GAPDH gene serving as an internal control. The relative expression of the target genes was calculated with the comparative threshold cycle (CT) method using the formula 2^−ΔCT^. Fold changes of target genes were calculated using the formula 2^−ΔΔCT^. The primers used for RT-qPCR are listed in **Supplementary Table 2**.

### Western Blot Analysis

Western blot analyses were performed to evaluate target protein expression. Briefly, protein lysates (~20 μg per sample) were loaded and run on 10% sodium dodecyl sulfate (SDS)-polyacrylamide gels, transferred to NC membranes (Millipore Corporation, MA, USA), and blocked with 5.0% nonfat milk for 3 h. The membranes were incubated with primary antibodies overnight at 4°C and then washed with Tris-buffered saline Tween-20 (TBST) three times for 10 min. Afterwards, membranes were incubated with the secondary antibodies for 2 h at room temperature and washed again. The bands on the membranes were visualized using an ECL system (Beyotime Institute of Biotechnology). Finally, membranes were exposed with an Odyssey infrared imaging system (LI-COR Biosciences, NE, USA). The following antibodies were used: anti-human FOLR1 (1:5000 dilution; Abcam, Cambridge, UK; CAT# ab221543), anti-human PCFT (1:300 dilution; Santa Cruz Biotechnology, Dallas, TX; CAT# sc-393460), anti-human RFC (1:300 dilution; Santa Cruz Biotechnology; CAT# sc-390948), anti-human GAPDH (1:5000 dilution; Abcam; CAT# ab181602), and HRP-labeled secondary antibodies (1:5000 dilution; Proteintech Group, Rosemont, IL).

### Immunofluorescence

A549 cells were fixed with 4% formaldehyde for 10 min, washed with PBS, and permeabilized with 0.1% Triton X-100 in PBS for 20 min. After washed with PBS and blocked with 5% BSA in PBS for 30 min, cells were incubated with primary antibody (anti-human FOLR1, 1:500 dilution) in 1% BSA-PBS overnight at 4°C, washed again, and incubated with fluorescent dye-conjugated secondary antibody for 1 h in the dark. Then, cells were washed with PBS for 3 times. Nuclei were stained with 4,6-diamidino-2-phenylindole (DAPI). Cells were analyzed by confocal microscopy (Zeiss, Stuttgart, Germany).

### Drug Treatment and Cell Viability Assay

For FRα induction, cells were cultured in medium supplemented with different concentrations of decitabine (0.1, 0.5, 2.5, 5 or 10 μM) for 72 h, or cells were treated with decitabine for different time (24, 48, 72 or 96 h). The medium was refreshed daily. Finally, total RNA or proteins were extracted. Cell viability was evaluated using the cell counting kit-8 (CCK-8) assay (MedChemExpress, NJ, USA) as described previously (12). Briefly, cells were seeded in 96-well plates at a density of 2000 cells/well. The medium was refreshed with drug-containing medium every 24 h. After 96 h, the cell survival rate was assessed. The absorbance was measured at 450 nm using a Multiskan Spectrum (Thermo Fisher Scientific, IL, USA). A concentration range of 0.005-2.0 μM pemetrexed and 0.05-20 μM decitabine was used to determine the IC_50_ values. The IC_50_ values of the concentration-response curves were calculated using CompuSyn software developed by Chou (21). Interactions between pemetrexed and decitabine were evaluated using CalcuSyn software and presented as the combination index (CI): CI < 1 indicates synergism, CI = 1 indicates an additive effect, and CI > 1 indicates antagonism.

### Pemetrexed Accumulation and LC-MS/MS Assay

Cells were seeded in 24-well plates to perform pemetrexed accumulation studies. Cells were washed with fresh medium once upon reaching 80% confluence, and then cells were incubated with drug-containing medium (50 μM pemetrexed) for 2, 4, 6, or 8 h at 37°C. Thereafter, the cells were gently washed with cold PBS 3 times and lysed with 0.1% SDS buffer. The pemetrexed concentration was quantified using a 4500 triple quadrupole mass spectrometer (AB Sciex, CA, USA) with a Turbo Ion Spray probe in positive mode. Multiple reaction monitoring (MRM) mode was used for quantitation based on the transitions of protonated molecular ions of pemetrexed at m/z 428.3−281.1 and anlotinib (internal standard) at m/z 408.3−304.2. The optimized mass spectrometric parameters were as follows: dwell time of 150 milliseconds, cell exit potential and entrance potential were 10 V and 12 V, respectively, collision energy (CE) of pemetrexed and anlotinib of27 V and 30 V, respectively, spray voltage temperature of 5500°C, and source temperature of 550°C. Data acquisition and processing were completed using Analyst software (version 1.6.2, AB Sciex, Framingham, MA). The chromatography analysis was performed using an LC-30AD system (Shimadzu Corporation, Kyoto, Japan) equipped with an ACQUITY UPLC BEH T3 column (2.1 mm×50 mm, 1.8 μm, Waters, USA) which was maintained at 40°C. A gradient elution program was conducted with mobile phase A (0.1% FA in 10 mM ammonium acetate/water) and mobile phase B (acetonitrile) as follows: 0 - 0.5 min, 90% of A; 0.5 - 1.5 min, 90% - 5% of A; 1.5 - 2.2 min, 5% of A; 2.3-3.8 min, 90% of A and finished at 3.8 min. The flow rate of the mobile phase was 0.4 mL/min. The injection volume was 5 μL.

### Animal Study

Six-week-old male BALB/c nude mice were purchased from the Experimental Animal Center of the Zhejiang Academy of Medicinal Sciences (Hangzhou Medical College, Hangzhou, China). All procedures were approved by the Institutional Animal Care and Use Committee of Zhejiang University. Mice were inoculated subcutaneously in the flank with 5 × 10^6^ A549 or A549R cells. When the volume of the formed tumor reached approximately 0.1 cm^3^, mice were intraperitoneally injected with 2.5, 5.0, or 10 mg/kg decitabine three times at intervals of 3 h on day 0, and tumors were excised on day 4 and 7 to extract total proteins for immunoblot analyses. A dose of 5.0 mg/kg decitabine that effectively induced FRα expression was chosen for subsequent experiments. Mice were randomly divided into 4 groups with 6 mice in each group: (1) control group (Control): saline; (2) decitabine group (DAC): 5 mg/kg decitabine; (3) pemetrexed group (PEM): 100 mg/kg pemetrexed; (D) pemetrexed plus decitabine group (Combo): 100 mg/kg pemetrexed and 5 mg/kg decitabine. Decitabine was administered intraperitoneally three times at intervals of 3 h (22) on day 1, 8, and 15, and pemetrexed was administered intraperitoneally on day 5, 12, and 19 (23). The tumor size was measured once every 4 days, and the volumes were calculated as follows: L×W^2^/2, where L and W are the longest and shortest dimensions, respectively.

### Quantification of Serum FRα Levels

The concentrations of the FRα protein in human serum were determined using a Human FOLR1 ELISA kit (R&D Systems, Minneapolis, USA) according to the manufacturer’s instructions.

### Statistical Analyses

GraphPad Prism version 8 for Windows (La Jolla, CA) was used to perform all statistical analyses, and data are expressed as the mean ± SE. When comparing two groups, Student’s *t*-test (unpaired two-tailed) was used. For multiple comparisons, one-way analysis of variance (ANOVA) was used followed by Dunnett’s or Tukey’s *post hoc* test. A probability of p < 0.05 was considered as statistically significant.

## Results

### Long-Term Fractionated Radiotherapy Downregulated the Expression of FRα in A549 Cells

**Figure 1:**
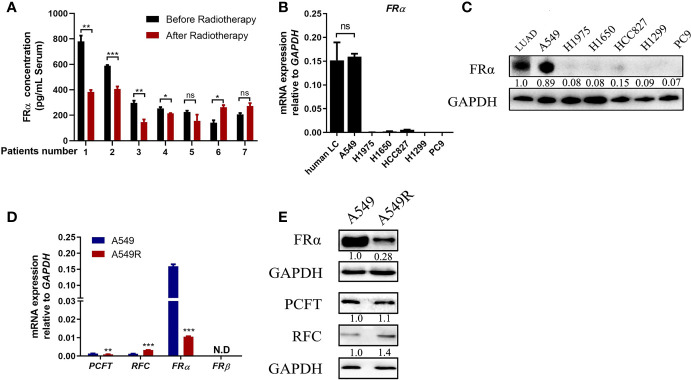
Long-term fractionated radiotherapy downregulated the expression of FRα in A549 cells. **(A)** Serum FRα concentration in 7 patients with LUAD. **(B)** The FRα mRNA expression level in human LUAD tissues and LUAD cell lines. **(C)** Expression of the FRα protein in human LUAD tumors (5 human tumors were pooled) and cell lines. **(D)** The mRNA expression of pemetrexed-related transporters and receptors. *PCFT* and *FRα* were significantly downregulated in A549R cells compared with A549 cells. *RFC* was upregulated and *FRβ* was not detected. **(E)** Expression of the FRα, PCFT and RFC proteins in A549 and A549R cells. Data are presented as the means ± SE, n =18 (human LUAD tissues) or 3 (cell lines or serum). Differences between A549 and A549R cells are denoted as *p < 0.05, **p < 0.01, and ***p < 0.001. ns, not significant. For immunoblots, densitometric values are shown as optical density after GAPDH normalization using Image J.

We collected serum samples from seven patients with LUAD before and after radiotherapy. These patients received an average dose of more than 50 Gy radiotherapy with a fractionated dose of 2-3 Gy and had not received any other traditional chemotherapy before or during radiotherapy. Five patients presented a significant reduction in serum FRα levels after radiotherapy (**Figure 1A**), suggesting that long-term fractionated radiotherapy may downregulate the expression of FRα. Therefore, we screened several commonly used LUAD cell lines, including NCI-H1975, NCI-H1650, NCI-HCC827, NCI-H1299, A549 and PC9 cells, to determine the proper cell lines to construct radioresistant cell models. Most LUAD cell lines exhibited limited expression of both the FRα mRNA and protein (**Figures 1B, C**), except for A549 cells, which had a comparable FRα expression level to human LUAD tissues, consistent with a previous report that A549 cells were FRα-positive cells among 19 LUAD cell lines (24). We constructed the acquired radioresistant LUAD cell line A549R by treating A549 cells with fractionated irradiation at 60 Gy and then examined the expression of pemetrexed related transporters and receptors. As shown in **Figure 1D**, *FRα* was abundantly expressed in A549 cells, while the expression levels of *RFC*, *PCFT*, and *FRβ* were relatively low. After long-term fractionated irradiation, the level of the FRα transcript decreased approximately 15-fold and the RFC transcript level increased approximately 2-fold in A549R cells compared with the parental cells. Similarly, the level of the FRα protein was significantly decreased by long-term fractionated irradiation (**Figure 1E**).

### A Low-Expression Level of FRα Decreased Pemetrexed Efficacy in A549R Cells

**Figure 2:**
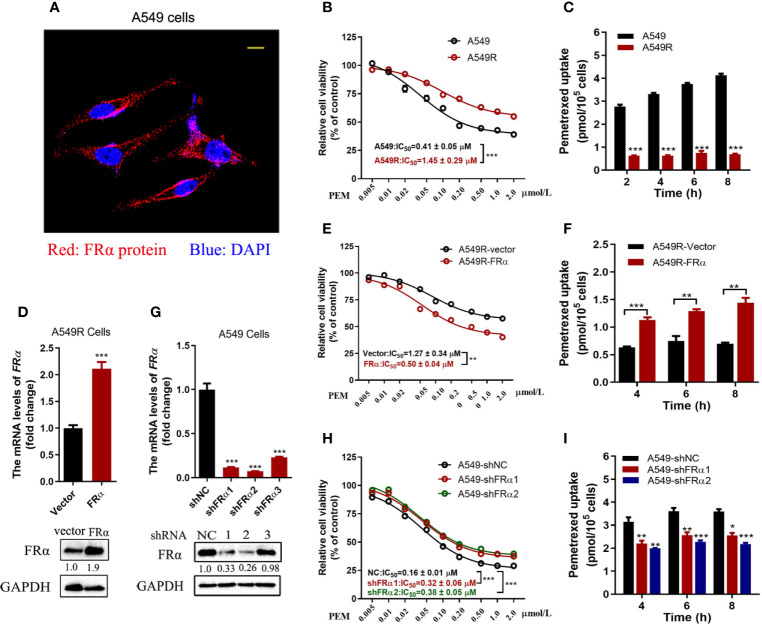
Low FRα expression decreased the efficacy of pemetrexed. **(A)** Confocal microscope image to determine the location of FRα in A549 cells. Bar = 10 μm. **(B, E, H)** Effect of pemetrexed on the viability of A549, A549R and their cell models. All cells were treated with pemetrexed at a concentration range of 0.005-2.0 μM for 96 h and then evaluated with the CCK-8 assay. Data are expressed as mean ± SE, n =5. **(D)** Cell models in which A549R cells stably overexpressing FRα or **(G)** A549 with stable knockdown of FRα. **(C, F, I)** Cellular accumulation of pemetrexed (50 μM) in A549, A549R and their cell models over 2, 4, 6 and 8 h. Data are presented as the means ± SE, n =3. Differences between A549 and A549R cells, A549R-vector and A549R-FRα cells, A549-shNC and A549-shFRα-1/shFRα-2 cells are denoted as: *p < 0.05, **p < 0.01, and ***p < 0.001, respectively.

FRα is abundantly expressed on the plasma membrane of A549 cells (**Figure 2A**) and facilitates pemetrexed to entry into cells ‘slowly’ through endocytosis. Notably, a high expression level of FRα exerts a substantial effect on the efficacy of pemetrexed; thus, we examined the IC_50_ values of pemetrexed in A549 and A549R cells. Cell viability was evaluated after cells were treated with pemetrexed for 96 h. The dose-response curve significantly shifted to the right from A549 to A549R cells, and the IC_50_ value was increased from 0.41 μM to 1.45 μM (**Figure 2B**). Meanwhile, pemetrexed accumulation was induced by incubating cells with 50 μM pemetrexed for 2, 4, 6, and 8 h, and the cellular contents of pemetrexed were detected using LC-MS/MS. The uptake study revealed that the cellular concentration of pemetrexed in A549 cells was 6-fold higher than that in A549R cells (**Figure 2C**). In addition, we excluded the possibility that the expression of pemetrexed target enzymes (including TYMS, DHFR, and GART) and folylpolyglutamate synthetase (FPGS), an important enzyme that increases the intracellular activity of pemetrexed (25), was perturbed by long-term fractionated irradiation (**Supplementary Figure 1A**).

Overexpression and knockdown experiments further validated our results that the low expression level of FRα led to decreased cytotoxicity of pemetrexed. As shown in **Figures 2D–F**, A549R cells overexpressed with FRα, the dose-response curve shifted to the left significantly from A549R-vector to A549R-FRα cells, and the IC_50_ value was reduced from 1.27 μM to 0.5 μM (**Figure 2E**). Additionally, the intracellular pemetrexed concentration was increased in A549R-FRα cells (**Figure 2F**). Similar results were observed in H1299 cells with overexpression of FRα, the IC_50_ value was reduced from 0.29 μM to 0.08 μM (**Supplementary Figure 2**). A549 cells were stably transfected with shRNA to knockdown FRα (**Figure 2G**). The dose-response curve shifted to the right from A549-shNC to A549-shFRα1/2 cells, and the IC_50_ values were increased approximately 1-fold (**Figure 2H**). The intracellular pemetrexed concentration was decreased in A549 cells transfected with FRα-targeting shRNAs (**Figure 2I**). Based on these results, low FRα expression in cells decreased intracellular pemetrexed accumulation, thus reducing its cytotoxicity.

### Decitabine Partially Reversed FRα Expression in A549R Cells Through an Indirect Regulatory Mechanism

**Figure 3:**
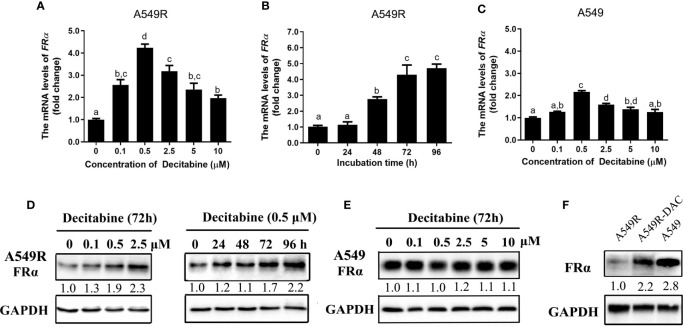
Decitabine upregulated the expression of FRα in A549R cells in a concentration-dependent and time-dependent manner. For the concentration-dependent study, cells were treated with different concentrations of decitabine (0.1-10 μM) for 72 h before RNA or protein was extracted. For the time-dependent study, cells were treated with 0.5 μM decitabine for 24, 48, 72 or 96 h before RNA or protein was extracted. **(A)** Decitabine upregulated *FRα* expression in A549R cells in a concentration-dependent manner. **(B)** Decitabine upregulated *FRα* expression in A549R cells in a time-dependent manner. **(C)** Decitabine slightly upregulated *FRα* expression in A549 cells. **(D)** Decitabine increased the expression of the FRα protein in a concentration-dependent and time-dependent manner. **(E)** Decitabine did not alter the expression of the FRα protein in A549 cells. **(F)** Levels of the FRα protein in A549, A549R and decitabine-treated A549R (A549R-DAC) cells that were exposed to 0.5 μM decitabine for 72 h before proteins were harvested. Data are presented as the means ± SE, n =3. Treatment groups with different letters shower significant differences using ANOVA followed by Tukey’s test. For immunoblots, densitometric values are shown as optical density after GAPDH normalization using Image J.

We analyzed the expression of FRα-related transcription factors to explore the possible mechanisms underlying the downregulation of FRα and found no difference in transcript levels between A549 and A549R cells (**Supplementary Figure 3**). Interestingly, when A549R cells were treated with decitabine, a demethylating reagent that blocks cellular DNA methyltransferases (DNMTs) (26), the expression of FRα was significantly upregulated. In addition, decitabine upregulated the expression of the FRα transcript in a concentration-dependent (**Figure 3A**) and time-dependent manner (**Figure 3B**) and the optimal concentration of decitabine was 0.5 μM. Similar results were observed for protein expression, as the level of the FRα protein was increased in A549R cells treated with increasing decitabine concentrations and treatment times (**Figure 3D**). However, decitabine did not affect FRα expression in A549 cells (**Figure 3E**) but weakly upregulated the expression of its mRNA (**Figure 3C**). Notably, although decitabine exerted a strong effect on reversing the change in FRα expression in A549R cells, it did not cause the expression of FRα to reach the original level (**Figure 3F**), indicating that decitabine only partially reversed the change in FRα expression.

**Figure 4:**
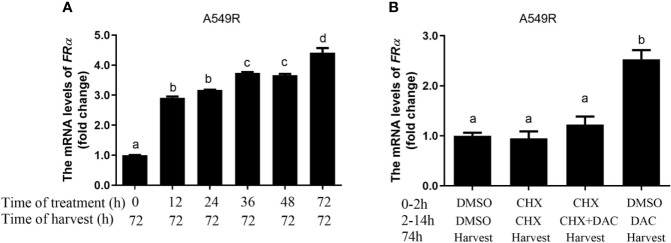
Decitabine delayed the induction of *FRα* expression in A549R cells. **(A)** The fold change in the expression of the FRα mRNA in A549R cells treated with 0.5 μM decitabine for the indicated periods. After decitabine treatment, cells were washed with decitabine-free media and replenished with new media that did not contain decitabine. At 72 h, all cells were collected for mRNA extraction. **(B)** A549R cells were pretreated with cycloheximide (CHX; 10 μM) followed by decitabine (DAC; 0.5 μM) as indicated. Twelve hours later, the cells were washed with decitabine/cycloheximide - free media and replenished with new media that did not contain decitabine/cycloheximide. All cells were harvested 60 h later, and total RNA was extracted for the quantification of the FRα mRNA by using RT-qPCR. Data are presented as the means ± SE, n =3. Treatment groups with different letters show statistically differences using ANOVA followed by Tukey’s test.

Decitabine was used to globally inhibit DNA methylation in LUAD cells, thus we speculated that long-term fractionated radiotherapy might induce aberrant methylation in the promoter region of FRα, resulting in low FRα expression. Bisulfite sequencing PCR (BSP) was used to examine methylation of 15 CG sites (-2599 bp to -2152 bp), which contain the only one CpG island in the promoter region of the *FOLR1* gene (27). All CG sites were hypermethylated, and no significant difference in methylation was observed between A549 and A549R cells (**Supplementary Figure 4**). Another interesting phenomenon was the delayed induction of *FRα* expression by decitabine, which began between 24 h to 48 h and reached up to a 4-fold elevation at 96 h (**Figure 3B**). In addition, when decitabine was withdrawn at 12, 24, 36, and 48 h, the expression of the FRα mRNA further increased, as observed at 72 h (**Figure 4A**). This delayed regulation of *FRα* expression by decitabine suggests that this process is likely mediated indirectly through other productions of decitabine. Cycloheximide was used to inhibit *de novo* protein synthesis in the early stage (0-12 h) of decitabine treatment to confirm this hypothesis (**Figure 4B**). The delayed induction of the FRα mRNA observed at 72 h after only a 12-h treatment with decitabine was abrogated when cycloheximide was added (**Figure 4B**). These results indicated that decitabine reversed the change in FRα expression in A549R cells through indirect regulation by inducing the *de novo* synthesis of some other protein(s) instead of directly acting on the promoter of FRα.

### Cytotoxic Synergism of Pemetrexed and Decitabine

**Figure 5:**
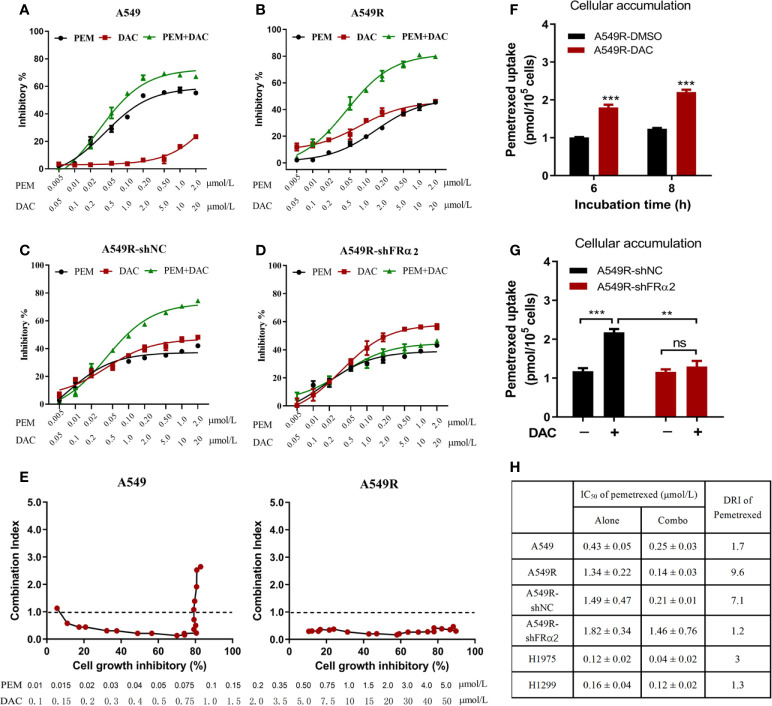
Decitabine enhanced the cytotoxicity of pemetrexed *in vitro*. **(A–D)** A549, A549R, A549R-shNC and A549R-shFRα cells were treated with pemetrexed (PEM) and decitabine (DAC) alone or in combination, at the indicated concentrations for 96 h. The results are expressed as the percentage of surviving drug-treated cells relative to DMSO-treated control cells. **(E)** Combination index-fraction affected plots of pemetrexed and decitabine combinations in A549 and A549R cells. The concentration ranges of pemetrexed and decitabine are 0.01-5 and 0.1-50 μmol/L. A cytotoxicity index (CI) < 1, CI = 1, and CI > 1 indicates synergism, an additive effect, and antagonism, respectively. **(F)** Effects of decitabine on pemetrexed cellular accumulation. DMSO-treated and decitabine-treated (0.5 μM, 96h) A549R cells were incubated with 50 μM pemetrexed for 6 or 8 h. **(G)** Effects of FRα knockdown on pemetrexed cellular accumulation. DMSO-treated and decitabine-treated (0.5 μM, 96h) cells were incubated with 50 μM pemetrexed for 6 h. **(H)** IC_50_ values and dose reduction index (DRI) of pemetrexed in cells receiving pemetrexed alone or combination treatment. DRI is the ratio of IC_50_ (alone) to IC_50_ (combo). **(A–D, H)**, data are presented as the means ± SE, n =5. E and F, data are presented as the means ± SE, n =3. Differences between cells are denoted as **p < 0.01 and ***p < 0.001. ns, not significant.

Next, the cytotoxicity of pemetrexed and decitabine alone or in combination was assessed. Based on the IC_50_ values of pemetrexed and decitabine in A549R cells, a molar ratio of pemetrexed:decitabine of 1:10 was used for the drug combination studies. A549 cells treated with pemetrexed concurrently with decitabine exhibited increased cytotoxicity (**Figure 5A**). However, the combination index values increased as the concentrations of pemetrexed and decitabine increased and finally exceeded 1.0, indicating that the combination of high concentrations of pemetrexed and decitabine resulted in an antagonistic effect (**Figure 5E**). On the other hand, the growth inhibition effect of the combination of pemetrexed and decitabine was more potent than that of pemetrexed or decitabine alone in A549R cells (**Figure 5B**), with the sensitivity of A549R cells to pemetrexed increasing by 9.6-fold (**Figure 5H**). All the combination index (CI) values were less than 1.0 and consistently remained at low values as the cell growth inhibitory fraction increased (**Figure 5E**).

To further determine whether the synergistic effect depends on FRα activation by decitabine, we assessed the efficacy of combination therapy in A549R-NC and A549R-shFRα cells (**Figures 5C, D**). Drug combination analysis showed that FRα shRNA expression markedly inhibited the sensitivity of A549R cells to pemetrexed by DAC treatment (**Figure 5H**). In addition, cellular accumulation studies showed decitabine increased the concentration of pemetrexed in A549R cells (**Figure 5F**) and this concentration increase could be attenuated by shFRα (**Figure 5G**). We also assessed the synergistic effect of combination therapy on H1299 and H1975 cells that have low expression levels of FRα. Drug combination analysis showed there was no or a weak synergistic effect of pemetrexed and decitabine on H1299 cells and H1975 cells (**Supplementary Figure 5** and **Figure 5H**).

In summary, these results present a better cytotoxic synergistic effect of pemetrexed and decitabine on A549R cells than on other LUAD cells, and suggest decitabine sensitizes A549R cells to pemetrexed mainly through upregulation of FRα.

### Decitabine Enhanced the Cytotoxicity of Pemetrexed *In Vivo*

**Figure 6:**
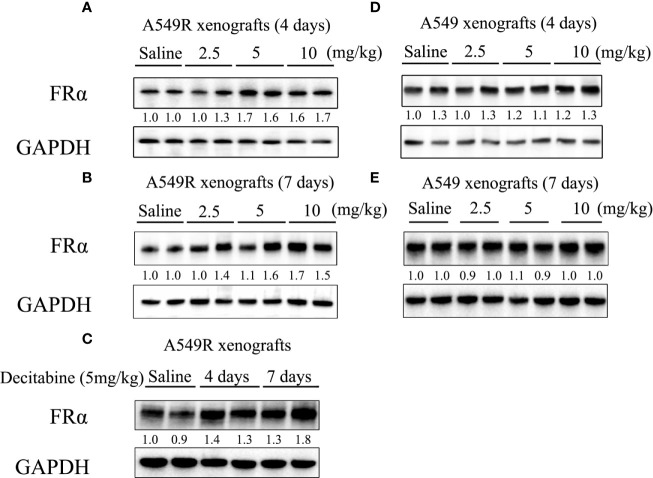
FRα protein expression in xenograft tumors from mice treated with decitabine. **(A, B)** FRα expression in A549R xenograft tumors. Mice were intraperitoneally injected with different doses (2.5, 5, or 10 mg/kg) of DAC on day 0, and tumors were collected on days 4 and 7. **(C)** Comparison of FRα expression in A549R xenograft tumors collected on day 4 and 7. **(D, E)** FRα expression in A549 xenograft tumors. Mice were intraperitoneally injected with different doses (2.5, 5, or 10 mg/kg) of DAC on day 0, and tumors were collected on day 4 and 7. Densitometric values are shown as optical density after GAPDH normalization using Image J and each band represents a mouse.

Based on the results described above, studies were performed to investigate the cytotoxic synergism of pemetrexed and decitabine *in vivo* by establishing A549 and A549R xenografts. First, we explored the effective concentration and treatment time of decitabine *in vivo*. Mice were intraperitoneally injected with different doses (2.5, 5 or 10 mg/kg) of decitabine on day 0 and tumors were collected on day 4 and 7. Treatment with 5 mg/kg and 10 mg/kg decitabine significantly upregulated FRα expression in A549R tumors on day 4 (**Figure 6A**), and this upregulation was more obvious on day 7 (**Figures 6B, C**). However, we did not observe the induction of FRα by decitabine in A549 xenografts (**Figures 6D, E**).

**Figure 7:**
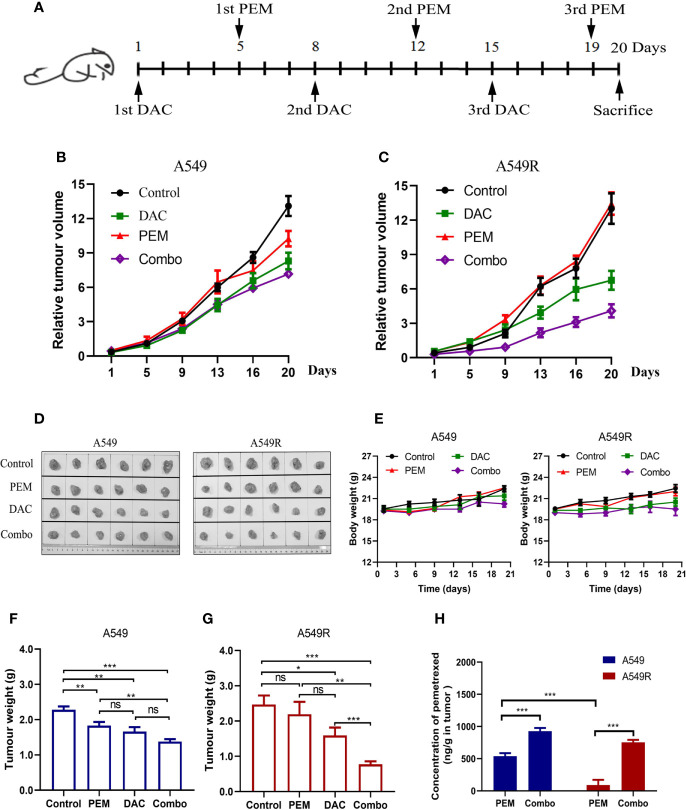
Decitabine sensitizes A549 and A549R cells to pemetrexed in xenograft models. **(A)** Experimental timeline and dosing schedule for xenograft models. **(B, C)** Tumor growth curves for A549 and A549R xenografts. **(D)** Tumors after resection from mice on day 20. **(E)** Body weight curves for A549 and A549R xenografts. **(F, G)** Tumor weight of A549 and A549R xenografts on day 20. **(H)** The concentration of pemetrexed in tumors of A549 and A549R xenografts. Data are presented as the means ± SE from 6 mice. Groups with different letters show significant differences using ANOVA followed by Tukey’s test. Differences between the two groups are denoted as *p < 0.05, **p < 0.01 and ***p < 0.001. ns, not significant. Control, DAC, PEM, and Combo indicate mice treated with saline, decitabine alone, pemetrexed alone, and decitabine-pemetrexed combination, respectively.

The drug administration timeline is shown in **Figure 7A**. Mice were treated with 100 mg/kg pemetrexed after injection of 5 mg/kg decitabine for 4 days. As showed in **Figure 7B**, compared to the saline-treated group, the tumor volume of A549 xenografts was decreased in the pemetrexed-treated group, but pemetrexed did not reduce the tumor volume of A549R xenografts (**Figure 7C**), consistent with the previous result that A549R cells were less sensitive to pemetrexed. As expected, the tumor volume in the combination group was significantly decreased in both A549 and A549R xenografts (**Figure 7D**) and similar results were observed for the tumor weight (**Figures 7F, G**). Moreover, the combination of pemetrexed and decitabine showed stronger growth inhibitory effects on A549R xenografts than on A549 xenografts. The concentrations of pemetrexed in tumors were detected by using LC-MS/MS, and the results indicated that decitabine increased the amount of pemetrexed by 2-fold in A549 tumors and 7-fold in A549R tumors (**Figure 7H**). Interestingly, although decitabine did not induce FRα expression in A549 tumors, the amount of pemetrexed in tumors of the combination group was also increased. By analyzing the levels of pemetrexed-related transporters, we found that the expression of *RFC* and *PCFT* was increased significantly (**Supplementary Figure 6**), which might account for the increased pemetrexed concentration detected in A549 tumors of the combination group. In addition, in the pemetrexed-treated group, the amount of pemetrexed in A549 tumors was much greater than that in A549R tumors, which explained the weaker growth inhibitory effect of pemetrexed on A549R xenografts (**Figure 7H**). H&E staining shows neither nephrotoxicity nor hepatotoxicity in mice undergoing different treatments (**Supplementary Figure 7**) and no significant weight loss was observed in each group (**Figure 7E**). Collectively, these results further indicated that decitabine enhanced the cytotoxicity of pemetrexed in A549R cells mainly by upregulating the expression of FRα *in vivo*.

## Discussion

Many studies have focused on the mechanisms of radioresistance and the discovery of radiosensitizers. However, limited information is available on changes in the efficacy changes of chemotherapeutic drugs during long-term radiotherapy and, in particular, new drug strategies for patients with acquired radioresistance. In the present study, we showed that: 1) long-term fractionated irradiation downregulated FRα expression in LUAD cells, which directly decreased the intracellular accumulation of pemetrexed and resulted in low sensitivity of radioresistant cells to pemetrexed; 2) decitabine enhanced the cytotoxicity of pemetrexed *in vitro* and *in vivo* through the indirect upregulation of FRα; and 3) the combination of pemetrexed and decitabine showed strong synergetic cytotoxicity toward A549R cells, which might provide a new strategy for patients with LUAD presenting with acquired radioresistance.

FRα was reported to be expressed at high levels in LUAD (17, 18, 28). However, its expression level in LUAD cell lines is not consistent. According to our study, only the A549 cell line was FRα-positive, and other LUAD cell lines had little or undetectable expression of FRα at the mRNA or protein level. This result was shown in another study in which the researchers analyzed the expression of FRα in 27 lung cancer cell lines, including 19 LUAD, 1 lung squamous cell carcinoma (SqCC), 1 lung adenosquamous carcinoma (ASC), 3 small cell lung cancer (SCLC) and 3 lung large cell carcinoma (LCC) cell lines, and only observed strong bands in the Western blots of A549 (LUAD), H647 (ASC), H460 (LCC) and SBC-5, KB (SCLC) cell lines, whereas other cell lines had faint or no bands, especially LUAD cell lines (24). Given the importance of pemetrexed in the clinical treatment of LUAD and the similar expression level of FRα to human LUAD, we constructed an A549 radioresistant cell model to study changes in the sensitivity of radioresistant cells to pemetrexed, which has been widely used in other studies to illustrate the internal mechanisms of acquired radioresistance (29, 30). Radioresistant isogenic cell models have been generated for many human cancer lines and have formed the basis for the identification of mechanisms of radioresistance (31, 32). When we searched the Gene Expression Omnibus (GEO) datasets for radioresistant cancer cells and FRα, we found that FRα was downregulated in radioresistant B-precursor acute lymphoblastic (ALL) tumor cells and the radioresistant squamous cell carcinoma cell line SCC61 (**Supplementary Figure 8**), suggesting that the downregulation of FRα in radioresistant cancer cells might be a universal phenomenon. Therefore, the efficiency of FRα-related chemotherapeutic drugs must be closely monitored in for patients with acquired radioresistance.

The membrane transport of pemetrexed in mammalian cells is mediated by several transporters and receptors. RFC, which belongs to the SLC19 family (*SLC19A1*), is widely distributed in various human tissues and functions as an anion exchanger, but its activity and folate-concentrating ability are limited in a low pH microenvironment (33, 34). Unlike RFC, PCFT which is encoded by *SLC46A1*, functions optimally at acidic rather than neutral pH. However, it is mainly expressed in the jejunum, duodenum, kidney, spleen, placenta, liver, and choroid plexus, but rarely in the lung (34). The folate receptors FRα and FRβ are high-affinity binding proteins that mediate the transport of pemetrexed through endocytosis. FRα is expressed at high levels in epithelial cells of the placenta, female reproductive organs, breast, lung and various human solid tumors (15, 35), whereas FRβ is mainly detected in hematopoietic tissues (35). Although FRα requires complex processes to facilitate pemetrexed to enter into cells, it has an affinity for preferred substrates more than 3 orders of magnitude greater than RFC (13, 36). In addition, various groups have documented high FRα expression in NSCLC (16, 17, 19, 37) and the results of our study also showed the highest expression level of FRα among the four transporters and receptors in A549 cells. Collectively, FRα may play a pivotal role in pemetrexed treatment of LUAD, and downregulation of FRα inevitably leads to the low sensitivity of radioresistant LUAD cells to pemetrexed.

Drug combinations are currently the mainstream treatment strategy for cancer chemotherapy, and may produce favorable outcomes, including 1) increasing the efficacy of the therapeutic agent, 2) maintaining the same efficacy to avoid toxicity, and 3) minimizing or slowing the development of drug resistance (21). Decitabine was approved by Food and Drug Administration (FDA) as an epigenetic drug for patients with acute myeloid leukemia (AML) and myelodysplastic syndrome (MDS) (38). Although the antitumor mechanisms of decitabine are unclear, low doses of decitabine are efficacious against hematological neoplasms, rather than high doses that induced rapid DNA damage and cytotoxicity (39). Remarkably, low doses of decitabine exert durable antitumor effects on hematological and epithelial tumor cells but are not toxic to normal cells (39). In recent years, several clinical trials have reported the potential of decitabine as a single agent and in combination with other chemotherapeutics for NSCLC (40–44). However, single epigenetic agents had limited effects; thus, the investigative focus now lies on combination therapies of epigenetically active agents with conventional chemotherapy. Patients with lung cancer who are not eligible for aggressive chemotherapy might benefit from epigenetic therapy due to the lower number of side effects (43). In our study, the cytotoxicity of pemetrexed in radioresistant adenocarcinoma cells was decreased approximately 3-fold compared to that in the parental cells, suggesting that 4 times the dose of pemetrexed was needed to maintain the same efficacy, which may undoubtedly increase toxic side effects. Interestingly, the combination of pemetrexed and decitabine effectively increased the accumulation of pemetrexed in radioresistant adenocarcinoma cells. Moreover, *in vivo* and *in vitro* studies indicated that the combination of pemetrexed and decitabine exerted a strong synergistic effect on H1975, A549 and A549R cells, consistent with preclinical studies showing that long-term and low-dose decitabine had remarkable chemotherapeutic potential for tumor therapy (40). According to a report, the mean maximum plasma concentration (Cmax) of decitabine in human is 64.8 - 77.0 ng/ml (0.28 μmol/L - 0.34 μmol/L) (45). In our study, a low dose of decitabine (0.2 μmol/L) increased pemetrexed sensitivity 1.2- and 3.1-fold in A549 and A549R cells, respectively, which suggests the good potential of a clinical combination of these two drugs. Unfortunately, the present study did not clarify the specific mechanism by which decitabine regulates the expression of FRα, but decitabine did not reduce CpG methylation in the FRα promoter, indicating that decitabine indirectly activated FRα expression through a mechanism that requires further study.

In conclusion, this study provides important basic data on the changes in pemetrexed-related transporters and receptors after long-term fractionated irradiation and suggests that the pemetrexed-decitabine combination is a promising treatment option that sensitizes radioresistant LUAD cells to pemetrexed by increasing the FRα-mediated accumulation of pemetrexed in cancer cells. By extension, we may provide a more individualized chemotherapy regimen for patients with LUAD presenting with acquired radioresistance. The FRα expression pattern in patients with LUAD acquired radioresistance is suggested to be closely monitored, thereby increased the survival of patients based on pemetrexed treatment. Of course, more preclinical studies are needed to optimize the safety and efficacy of pemetrexed-decitabine combination therapy.

## Data Availability Statement

The original contributions presented in the study are included in the article/**Supplementary Material**. Further inquiries can be directed to the corresponding authors.

## Ethics Statement

The studies involving human participants were reviewed and approved by the Internal Review and the Ethics Board of Affiliated Hangzhou First People’s Hospital, Zhejiang University School of Medicine. Written informed consent for participation was not required for this study in accordance with the national legislation and the institutional requirements. The animal study was reviewed and approved by The Institutional Animal Care and Use Committee of Zhejiang University.

## Author Contributions

SZ, SM, and YW conceptualized the study. YW and JH designed the study and wrote the manuscript. YW, JH, QW, and JZ performed the experiments. ZM, LZ, and BX contributed to the editing of the manuscript. All authors contributed to the article and approved the submitted version.

## Funding

The work was supported by the Science and Technology Development Project of Hangzhou (grant. 20180533B98), Zhejiang Provincial Natural Science Foundation of China under Grant No. LQ19H310001 and LY19H160032, Zhejiang Provincial Medicine and Health Science Foundation (grant No. 2020RC027, 2020RC028) and National Natural Scientific Foundation of China (81803631).

## Conflict of Interest

The authors declare that the research was conducted in the absence of any commercial or financial relationships that could be construed as a potential conflict of interest.
