# Supplementary material for: Decitabine Sensitizes the Radioresistant Lung Adenocarcinoma to Pemetrexed Through Upregulation of Folate Receptor Alpha
Source: Front Oncol. 2021 May 17;11:668798. doi: 10.3389/fonc.2021.668798 (PMC8165306; doi:10.3389/fonc.2021.668798)
Supplement: Supplementary file 1 [file DataSheet_1.pdf]

Supplementary Material

1 Supplementary Tables

Table 1. Information of patients with LUAD

Number	Gender	Age	Sample type	Stage
1	Female	56	Tissue	I A
2	Male	75	Tissue	I A
3	Female	79	Tissue	I A
4	Female	53	Tissue	I A
5	Female	61	Tissue	I A
6	Female	68	Tissue	II B
7	Male	72	Tissue	I B
8	Female	79	Tissue	I A
9	Female	70	Tissue	IV A
10	Female	56	Tissue	I A
11	Male	50	Tissue	I A
12	Female	69	Tissue	II A
13	Male	47	Tissue	III A
14	Female	46	Tissue	I A
15	Male	65	Tissue	I A
16	Male	66	Tissue	II A
17	Male	68	Tissue	III A
18	Male	70	Tissue	II A
Number	Gender	Age	Sample type	Stage/Radiation dose
1	Female	61	Serum	IV B; 30Gy
2	Male	86	Serum	III B; 48Gy
3	Female	71	Serum	II A; 60Gy
4	Female	68	Serum	II A; 60Gy
5	Male	68	Serum	III A; 60Gy
6	Male	69	Serum	III B; 55Gy
7	Female	91	Serum	II B; 48Gy

Table 2. Primers and shRNAs used in this study

Name	Forward	Reverse
PCFT	GGGAAAGAGCCTTCAGCAG	GAAAGCACCCTCCAATAAACC
RFC	GGGCTTTGTTGCTGGAAG	GGCAGAAAGGATTTGTCTCAAG
FRα	TTAGCCTGGCCCTAATGCT	GCAGGGATTTCCAGGTATCA
FRβ	CAGCAACGGAGGTTCAGC	CATGGTGGCTACACAGACCA
TYMS	CCCAGTTTATGGCTTCCAGT	GCAGTTGGTCAACTCCCTGT
DHFR	GGAAGAATCGGCTCAAAACC	CTTGCCCTGCCATGTCTC
GART	TCCCTGAGAAACTTGGGGTA	GCTGCAACCATGAGAAGACC
FPGS	CCCTGCCAGTTTGACTATGC	CTGTGAAGTTCTGTTGGTCTGC
GR	ACAGCATCCCTTTCTCAACAG	AGATCCTTGGCACCTATTCCAAT
AR	TTGTGTCAAAAGCGAAATGG	AGTCAATGGGCAAAACATGG
PR	GTCAGTGGGCAGATGCTGTA	TGCCACATGGTAAGGCATAA
ERα	ATGATCAACTGGGCGAAGAG	CAGGATCTCTAGCCAGGCAC
ERβ	TCCATCGCCAGTTATCACATCT	CTGGACCAGTAACAGGGCTG
GAPDH	GCACCGTCAAGGCTGAGAAC	TGGTGAAGACGCCAGTGGA
Name	shRNA (5’- 3’)	
shFRα-1	GCTTCTCAATGTCTGCATGAATTCAAGAGATTCATGCAGACATTGAGAAGCTTTTTT	
shFRα-2	GGATGTTTCCTACCTATATTTCAAGAGAATATAGGTAGGAAACATCCTTTTTT	
shFRα-3	GCAATGAAATCTGGACTCACTTTCAAGAGAAGTGAGTCCAGATTTCATTGCTTTTTT	

2 Supplementary Figures

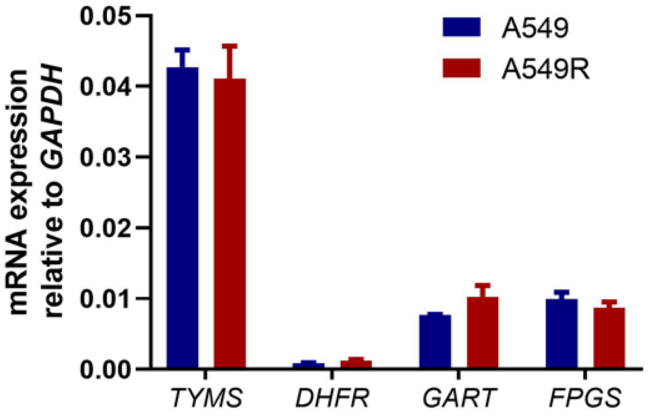

Supplementary Figure 1. The mRNA expression levels of pemetrexed-related enzymes in A549 and A549R cells. TYMS, thymidylate synthase; DHFR, dihydrofolate reductase; GART, glycinamide ribonucleotide formyltransferase; FRGS, folylpolyglutamate synthetase. Data are presented as the means ± SE, n =3.

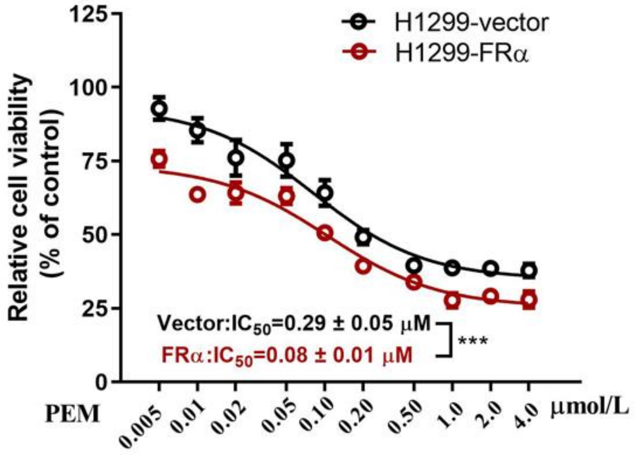

Supplementary Figure 2. Effect of pemetrexed on the viability of H1299-vector and H1299-FRα cells. H1299 cells were stably overexpressing FRα and treated with pemetrexed at concentrations ranging from 0.005 - 4.0 μM for 96 h and then evaluated with the CCK-8 assay. Data are presented as the means ± SE, n =5. Differences between the IC_{50} values are denoted as ***$p < 0.001$

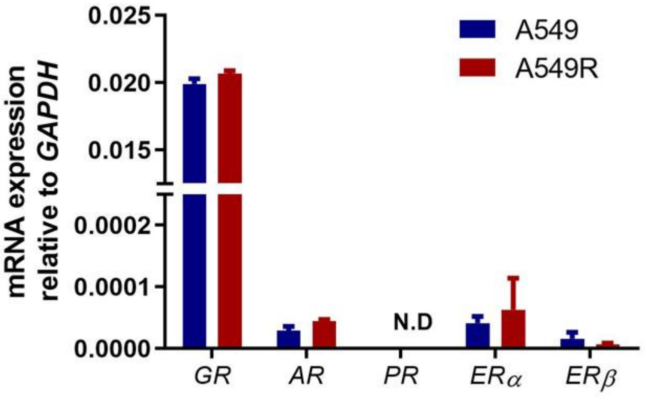

Figure 3 The mRNA expression levels of FRα-related transcription factors. GR, glucocorticoid receptor; AR, androgen receptor; PR, progesterone receptor; ERα, estrogen receptor α; ERβ, estrogen receptor β. Data are presented as the means ± SE, n =3.

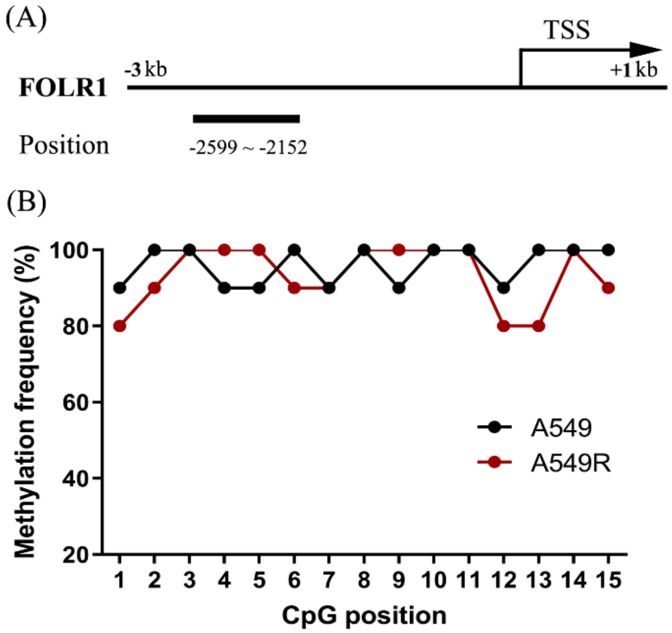

Supplementary Figure 4. DNA methylation analysis of *FOLR1* promoter in A549 and A549R cells. (a) Schematic diagram of 4 kb around *FOLR1* TSS. CpG island is included in the bisulfite sequencing region from position -2599 bp to -2152 bp. TSS, transcription start site. (b) The methylation frequency of *FOLR1* promoter in A549 and A549R cells. The methylation frequency of each CpG site is evaluated by calculating the methylated CpG proportion from 10 clones. The X-axis represents the individual 1~15 CpG sites.

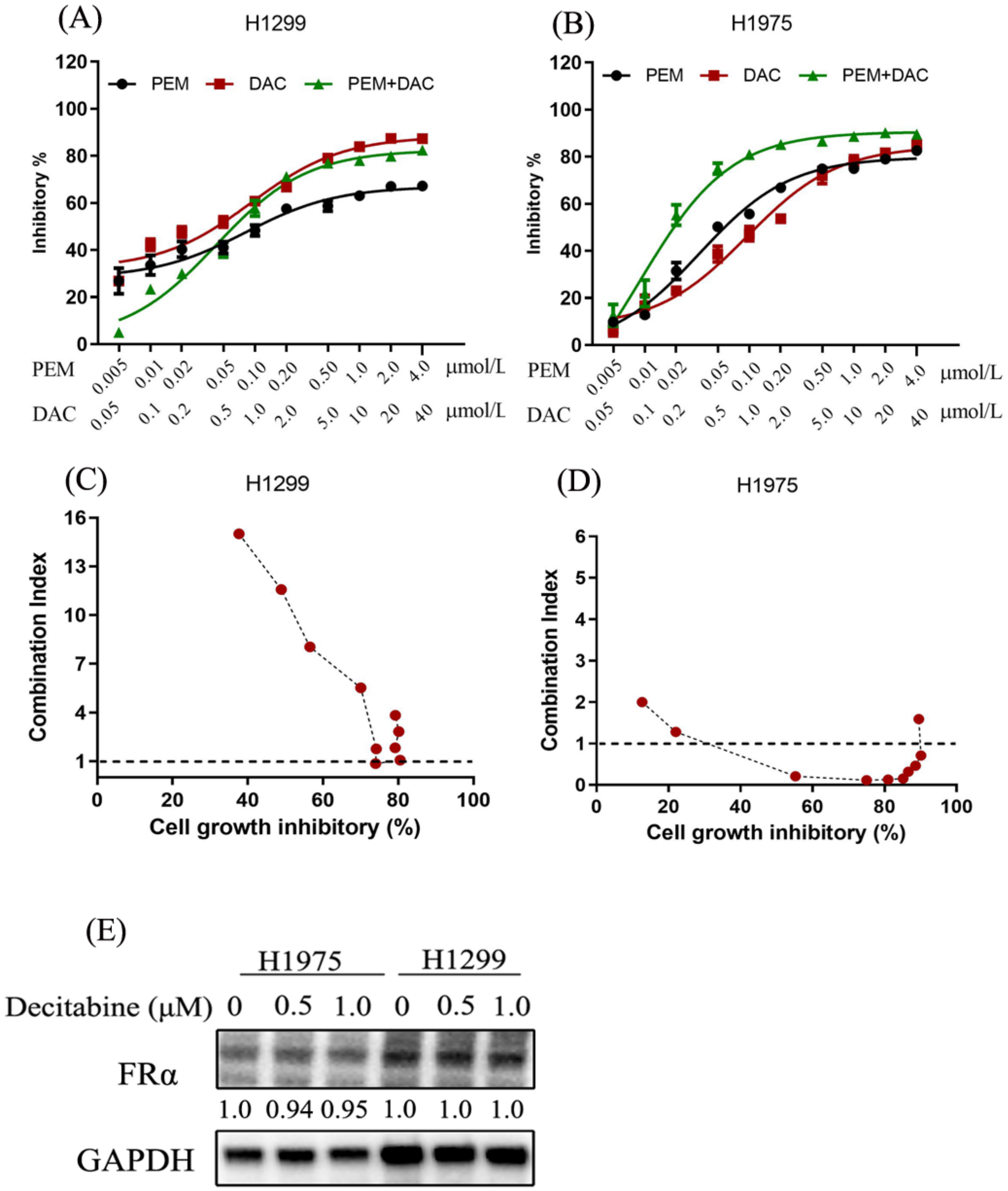

Supplementary Figure 5. Cytotoxic synergism effect of pemetrexed and decitabine on H1299 and H1975 cells. (A and B) H1299 and H1975 cells were treated with pemetrexed (PEM) and decitabine (DAC) alone or in combination at the indicated concentrations for 96 h. The results are reported as the percentage of surviving drug-treated cells relative to DMSO-treated control cells. Data are presented as the means ± SE, n =5. (C and D) Combination index-fraction affected plots of pemetrexed and decitabine combinations in H1299 and H1975 cells. The concentration ranges of pemetrexed and decitabine are 0.005-4 and 0.05-40 μmol/L. A cytotoxicity index (CI) < 1, CI = 1, and CI > 1 indicates synergism, an additive effect, and antagonism, respectively. (E) Decitabine did not alter the expression of the FRα protein in H1975 or H1299 cells. H1975 and H1299 cells were treated with 0, 0.5 or 1.0 μmol/L decitabine for 96 h and then total protein were extracted. Densitometric values are shown as optical density after GAPDH normalization using Image J.

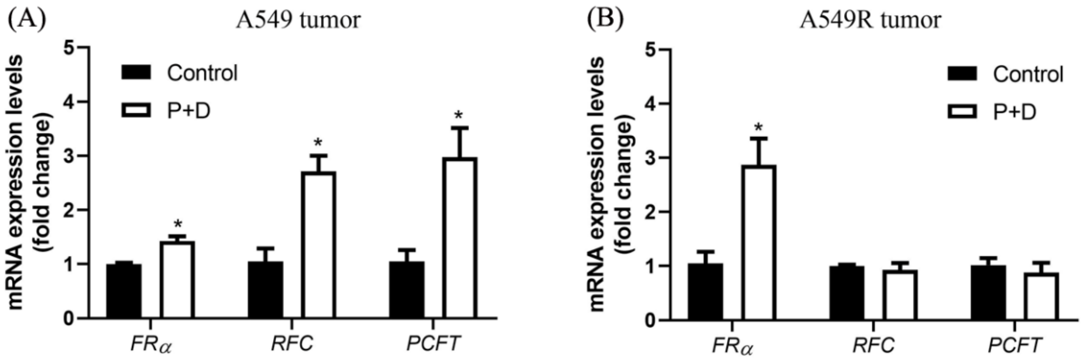

Supplementary Figure 6. mRNA fold change of *FRα*, *RFC* and *PCFT* in A549 and A549R xenograft tumors that were collected at the end of the tumor growth study after mice were treated with saline (Control) or the combination of pemetrexed and decitabine (P + D). (A) The mRNA expression levels of *FRα, RFC* and *PCFT* in A549 xenograft tumors of pemetrexed and decitabine combination (P + D) group were much higher than those in the Control group. (B) The mRNA expression levels of *FRα* in A549R xenograft tumors of pemetrexed and decitabine combination group was much higher than that in the Control group while *RFC* and *PCFT* had no significant difference. Data are presented as the means $\pm$ SE, n =3. Differences between the two groups are denoted as $^*p < 0.05$.

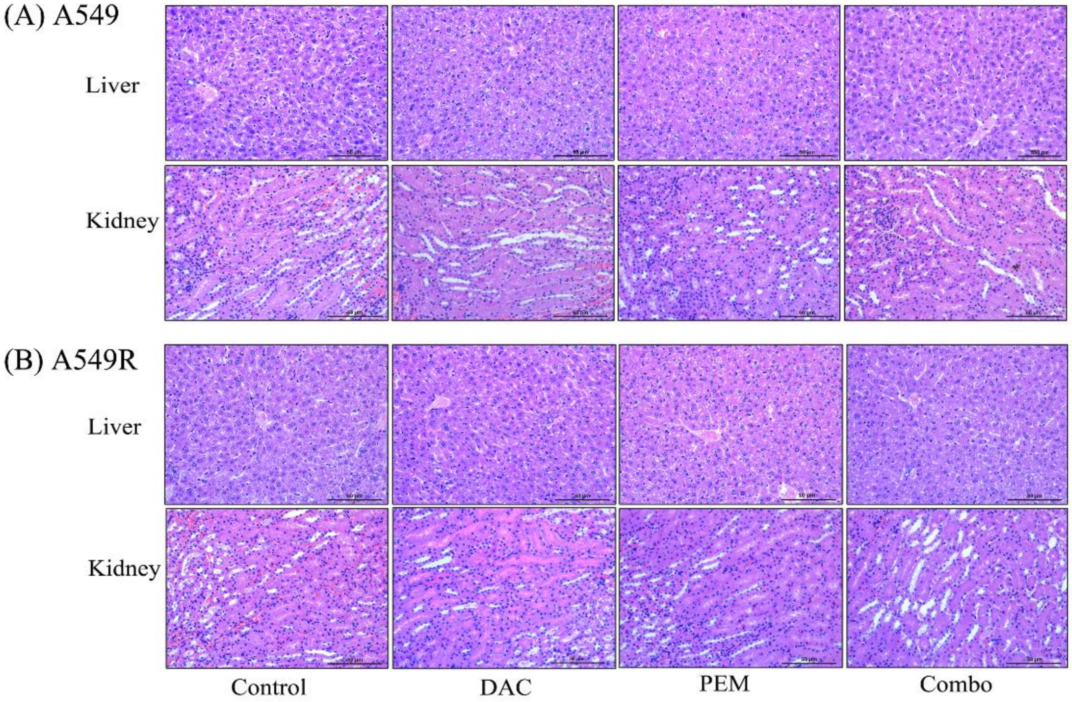

Supplementary Figure 7. Histological analysis was performed on kidney and liver sections collected from A549 and A549R xenografts. H&E staining shows neither nephrotoxicity nor hepatotoxicity in mice undergoing different treatments. Scale bar = 50 µm. Tissues were collected at the end of the tumor growth study. Control, DAC, PEM, and Combo indicate mice treated with saline, decitabine alone, pemetrexed alone, and combination of decitabine and pemetrexed, respectively.

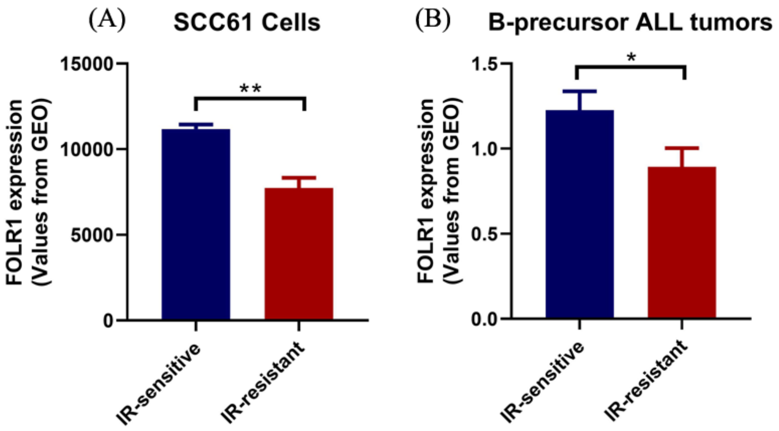

Supplementary Figure 8. Data from GEO that *FRα* (*FOLR1*) was downregulated in radioresistant squamous cell carcinoma cell line SCC61 and B-precursor acute lymphoblastic (ALL) tumor cells. (A) IR-resistant SCC61 cells were selected from a parental radiosensitive tumor SCC-61 by eight serial cycles of passage in athymic nude mice and in *vivo* irradiation. *FOLR1* was downregulated in IR-resistant cells. Data are expressed as the means ± SE, n = 3. GEO Profile, GDS3125 / 211074_at. (B) B-precursor acute lymphoblastic (ALL) tumor cells were exposed to ionizing radiation (IR) to induce DNA damage in vitro. *FOLR1* was downregulated in IR-resistant cells. Data are expressed as the means ± SE, n = 11. GEO Profile, GDS3471 / 204437_s_at. Differences between two groups are denoted as *$p < 0.05$, **$p < 0.01$.
